# Comorbidity burden may explain adiponectin’s paradox as a marker of increased mortality risk in hemodialysis patients

**DOI:** 10.1038/s41598-021-88558-0

**Published:** 2021-04-27

**Authors:** Ilia Beberashvili, Tamar Cohen-Cesla, Amin Khatib, Ramzia Abu Hamad, Ada Azar, Kobi Stav, Shai Efrati

**Affiliations:** 1 Nephrology Division, Yitzhak Shamir Medical Center, Zerifin, Affiliated with the Sackler Faculty of Medicine, Tel Aviv University, Zerifin, Israel; 2 Internal Department D, Yitzhak Shamir Medical Center, Zerifin, Affiliated with the Sackler Faculty of Medicine, Tel Aviv University, Zerifin, Israel; 3 Nutrition Department, Yitzhak Shamir Medical Center, Zerifin, Affiliated with the Sackler Faculty of Medicine, Tel Aviv University, Zerifin, Israel; 4 Urology Department, Yitzhak Shamir Medical Center, Zerifin, Affiliated with the Sackler Faculty of Medicine, Tel Aviv University, Zerifin, Israel

**Keywords:** Biomarkers, Nephrology

## Abstract

Despite experimental evidence of beneficial metabolic, antiatherosclerotic and antiinflammatory effects of the 30 kDa adipokine, adiponectin, maintenance hemodialysis (MHD) patients with high adiponectin blood levels have paradoxically high mortality rates. We aimed to examine the direction of the associations between adiponectin and all-cause and cardiovascular mortality as well as with markers of oxidative stress, inflammation and nutrition in MHD patients with varying degrees of comorbidities. A cohort of 261 MHD patients (mean age 68.6 ± 13.6 years, 38.7% women), grouped according to baseline comorbidity index (CI) and serum adiponectin levels, were followed prospectively for six years. High and low concentrations were established according to median CI and adiponectin levels and cross-classified. Across the four CI-adiponectin categories, the group with low comorbidities and high adiponectin exhibited the best outcomes. Conversely, the high comorbidity group with high adiponectin levels had the lowest survival rate in both all-cause mortality (log rankχ^2^ = 23.74, *p* < 0.001) and cardiovascular mortality (log rankχ^2^ = 34.16, *p* < 0.001). Further data adjustment for case-mix covariates including fat mass index did not substantially affect these results. In conclusion, the direction of adiponectin’s prognostic associations in MHD patients is inverse in those with few comorbidities and direct in those with many comorbidities.

## Introduction

Adiponectin, an adipokine with very important roles in carbohydrate and lipid metabolism and vascular biology^1^, was first characterized in 1995 and still remains a paradoxical hormone. Despite its insulin-sensitizing^2^, anti-inflammatory^3^ and antiatherogenic properties^4^, described in experimental studies and in the general population, adiponectin has been reported to be positively associated with mortality in some populations such as congestive heart failure^5^, myocardial infarction^6^, elderly^7^, and end stage kidney disease (ESKD) patients receiving maintenance hemodialysis (MHD)^8–10^ or peritoneal dialysis (PD)^10^ treatment. This 30 kDa protein is mainly secreted by white adipose tissue in three different isoforms, each with a different biological activity: a low-molecular-weight trimer, a middle-molecular-weight hexamer, and high-molecular-weight isoform assembled from middle-molecular-weight oligomers^11,12^. Adiponectin exerts multiple biological effects throughout the body mediated by two major receptors, AdipoR1 and AdipoR2^13^.

Compared to the general population, serum adiponectin levels are elevated in chronic kidney disease (CKD) patients, mainly due to decreased kidney function, with the highest levels in ESKD patients^14^. In MHD patients, similar to the general population, adiponectin maintains inverse associations with adiposity^14^, insulin resistance^15^, markers of oxidative stress^16^, and inflammation^17^. These markers are also known to be associated with morbidity and mortality in MHD patients^18–20^, and the direction of these relationships should result in adiponectin to be inversely related to adverse outcomes as well. However, regarding the association between serum adiponectin levels and survival in MHD patients, observational studies have shown conflicting results, with adiponectin levels reported to be associated with increased risks of mortality in most^8–10,17,21^ but not all studies^22,23^. In search for potential explanations of these contradictory results, some studies investigated potential confounder (such as body mass index (BMI), C-reactive protein (CRP), and N-terminal pro-B-type natriuretic peptide (NT-pro-BNP)) impacts on the association of adiponectin with outcomes by applying a stepwise approach^17^. Others found the modification effect of obesity on adiponectin’s association with all-cause mortality in MHD patients^24^. Despite these attempts, the factor(s) that differentiate the aforementioned study populations and may also lead to a reversal of the direction of association between adiponectin and mortality in MHD patients has not yet been identified. However, the common denominator of the populations in which the adiponectin paradox is described is that of high levels of comorbidities. We hypothesized that varying degrees of comorbidities may affect the association between adiponectin and mortality in MHD patients. The aim of the present study was therefore to examine the direction of the associations between adiponectin and all-cause and cardiovascular disease (CVD) related mortality as well as with markers of oxidative stress, inflammation and nutrition in MHD patients with varying degrees of comorbidities.

## Results

The median level of the comorbidity index (CI) for the 261 MHD patients at the start of the cohort was 4.0, with an interquartile range (IQR) of 2.0 to 7.0 and the median level of serum adiponectin was 8.28 mcg/ml, with an IQR of 3.84 to 32.68 mcg/ml. The patients were grouped according to CI and serum adiponectin levels. High and low concentrations were established according to median CI and adiponectin levels and cross-classified. The demographic, clinical and biochemical characteristics of the participants according to this categorization are detailed in Table 1. Patients with higher CI were older, mostly men, and smokers. They also had a higher prevalence of diabetes mellitus (DM), higher neutrophil to lymphocyte ratio (NLR) levels, along with higher interleukin-6 (IL-6), F2-isoprostanes (F2-IsoP), and waist circumference levels and lower uric acid and phase angle compared to the low CI group. Patients with high adiponectin were mostly men, had lower dialysis vintage, Kt/V, creatinine, CRP, fat mass index (FMI), acyl-ghrelin and F2-IsoP. However, they had higher CI and tumor necrosis factor-alpha (TNF-α) compared to lower adiponectin group. The prevalence of comorbid conditions present at baseline in the study participants grouped according to comorbidity index and serum adiponectin levels is shown in Table 2. The prevalence of cardiovascular disease, particularly congestive heart failure, cerebrovascular disease, dysrhythmias and peripheral vascular disease, was substantially higher in the higher CI group. Out of cardiovascular disease peripheral vascular disease and non-coronary cardiac diseases (including pericarditis, endocarditis, myocarditis, heart valve replacement, and cardiac devices) were more common in the patients with high adiponectin.

**Table 1:** Demographic, clinical and biochemical characteristics of the study population (n = 261), grouped according to comorbidity index and serum adiponectin (mcg/ml) levels.

	CI ≤ 4 (n = 142)		CI > 4 (n = 119)		MANOVA^b^
	Low Ad^a^ (n = 78)	High Ad (n = 64)	Low Ad (n = 52)	High Ad (n = 67)	
**Demographic and clinical characteristics**					
Age (years)	65.3 ± 15.5	68.2 ± 15.4	71.3 ± 10.1	71.0 ± 11.2	CI
Gender (men/women)^c^	42/58	60/40	73/27	75/25	CI, Ad
Vintage (months)^d^	1.46 ± 0.48	1.10 ± 0.52	1.44 ± 0.45	1.11 ± 0.58	Ad
DM (yes)^c^	47.4	50.8	71.2	71.6	CI
Comorbidity index^d^	0.25 ± 0.24	0.30 ± 0.23	0.83 ± 0.10	0.86 ± 0.11	Ad, CI
Kt/V	1.43 ± 0.33	1.29 ± 0.31	1.35 ± 0.30	1.24 ± 0.25	Ad
Residual renal function (yes)^c^	50.0	59.3	47.8	60.0	NS
Smoking (yes)^c^	5.1	3.2	34.6	17.9	CI
Handgrip strength (kg)^d^	1.23 ± 0.29	1.19 ± 0.25	1.26 ± 0.21	1.22 ± 0.22	NS
Men	1.45 ± 0.20	1.32 ± 0.17	1.34 ± 0.15	1.29 ± 0.18	CI, Ad
Women	1.07 ± 0.23	1.00 ± 0.21	1.01 ± 0.20	0.98 ± 0.12	NS
**Blood analyses**					
Albumin (g/dl)	3.84 ± 0.32	3.76 ± 0.38	3.73 ± 0.43	3.75 ± 0.34	NS
Transferrin (mg/dl)	167.8 ± 36.7	166.6 ± 30.6	169.3 ± 34.1	172.2 ± 23.4	NS
Creatinine (mg/dl)	8.07 ± 2.10	7.03 ± 2.37	7.51 ± 2.08	6.64 ± 1.84	Ad
Uric acid (mg/dl)	6.09 ± 1.25	5.68 ± 1.12	5.65 ± 0.91	5.55 ± 1.21	CI
Cholesterol (mg/dl)	150.8 ± 37.1	150.3 ± 38.8	141.7 ± 33.8	143.3 ± 32.7	NS
Triglycerides (mg/dl)	163.4 ± 90.8	144.4 ± 74.8	138.0 ± 69.4	150.9 ± 93.5	NS
Hemoglobin (g/dl)	11.0 ± 1.3	10.9 ± 1.2	11.3 ± 1.3	10.9 ± 1.1	NS
NLR^d^	0.50 ± 0.22	0.43 ± 0.47	0.55 ± 0.25	0.55 ± 0.19	CI
CRP (mg/L)^d^	0.89 ± 0.49	0.75 ± 0.49	0.98 ± 0.45	0.84 ± 0.47	Ad
IL-6 (pg/ml)^d^	0.84 ± 0.46	0.88 ± 0.34	0.99 ± 0.47	1.06 ± 0.36	CI
TNF-α (pg/ml)^d^	1.31 ± 0.18	1.40 ± 0.16	1.30 ± 0.15	1.37 ± 0.15	Ad
Acyl-Ghrelin (pg/ml)^d^	2.17 ± 0.39	2.03 ± 0.39	2.13 ± 0.41	1.96 ± 0.44	Ad
Leptin (ng/ml)^d^	0.63 ± 0.85	0.59 ± 1.07	0.54 ± 0.70	0.70 ± 0.68	NS
Men	0.18 ± 1.08	0.43 ± 1.17	0.48 ± 0.73	0.57 ± 0.68	NS
Women	0.93 ± 0.47	0.82 ± 0.88	0.72 ± 0.59	1.04 ± 0.57	NS
F2-IsoP (ng/ml)^e^	2.63 ± 1.36	2.39 ± 0.54	3.24 ± 1.44	2.49 ± 0.81	CI, Ad
**Body composition analyses**					
BMI (kg/m^2^)	27.7 ± 5.6	26.4 ± 5.8	27.9 ± 5.4	27.0 ± 5.8	NS
WC (cm)	103.7 ± 15.0	100.2 ± 17.5	105.7 ± 12.4	107.1 ± 15.0	CI
ECW/TBW	0.38 ± 0.05	0.39 ± 0.07	0.39 ± 0.05	0.40 ± 0.05	NS
FMI (kg/m^2^)	10.2 ± 4.2	8.6 ± 4.1	9.3 ± 4.2	8.6 ± 4.5	Ad
LBMI (kg/m^2^)	17.5 ± 3.1	17.9 ± 2.7	18.5 ± 2.4	18.8 ± 2.7	NS
Phase angle (°)	5.0 ± 1.2	4.8 ± 1.4	4.6 ± 1.0	4.4 ± 1.5	CI
**Nutritional scores**					
MIS^d^	0.69 ± 0.32	0.77 ± 0.24	0.74 ± 0.31	0.74 ± 0.25	NS
GNRI	110.9 ± 12.3	106.3 ± 13.1	107.5 ± 12.6	107.5 ± 11.3	NS

CI, comorbidity index; NS, non-significant; Ad, adiponectin; DM, diabetes mellitus; NLR, neutrophil to lymphocyte ratio; CRP, C-reactive protein; TNF-α, tumor necrosis factor α; IL-6, interleukin-6; F_2_-IsoP, F_2_-isoprostanes; BMI, body mass index; WC, waist circumference; ECW/TBW, extra-cellular water to total body water ratio; FMI, fat mass index; LBMI, lean body mass index; MIS, malnutrition-inflammation score; GNRI, geriatric nutritional risk index.

^a^Low adiponectin was defined as adiponectin < 8.28 mcg/ml, the value below the median of distribution.

^b^Two-factor MANOVA. Significant (*p* < 0.05) effects are given for comorbidity index (CI), adiponectin (Ad), and the interaction of comorbidity index with adiponectin (CI × Ad).

^c^Assessed by χ^2^ test.

^d^Continuous variables that did not follow a normal distribution (dialysis vintage, comorbidity index, handgrip strength, TNF-α, CRP, IL-6, acyl-ghrelin, leptin and MIS) were log-transformed before their insertion into this model.

^e^F2-IsoP levels were randomly measured in 233 participants.

**Table 2:** Prevalence (%) of comorbid conditions at baseline in the study population (n = 261), grouped according to comorbidity index and serum adiponectin (mcg/ml) levels.

Comorbid conditions^a^	CI ≤ 4 (n = 142)		CI > 4 (n = 119)		MANOVA^c^
	Low Ad^b^ (n = 78)	High Ad (n = 64)	Low Ad (n = 52)	High Ad (n = 67)	
Ischemic heart disease	23.1	23.8	55.8	59.7	CI
Congestive heart failure	5.1	4.8	59.6	70.1	CI
Cerebrovascular disease	6.4	6.3	30.8	43.3	CI
Peripheral vascular disease	2.6	3.2	21.2	35.8	CI, Ad
Dysrhythmia	10.3	14.3	40.4	34.3	CI
Other cardiac disease	1.3	4.8	11.5	23.9	CI, Ad
Lung disease	2.6	11.1	32.7	26.9	CI
Gastrointestinal bleeding	3.8	0.0	9.6	7.5	CI
Liver disease	9.0	4.8	15.4	6.0	NS
Cancer	14.1	19.0	28.8	22.4	NS
Diabetes mellitus	47.4	50.8	71.2	71.6	CI

CI, comorbidity index; NS, non-significant; Ad, adiponectin.

^a^See Methods for definitions of comorbid conditions.

^b^Low adiponectin was defined as adiponectin < 8.28mcg/ml, the valuebelow the medianof distribution.

^c^Two-factor MANOVA. Significant (*p* < 0.05) effects are given for comorbidity index (CI), adiponectin (Ad), and the interaction of comorbidity index with adiponectin (CI × Ad).

**Figure 1:**
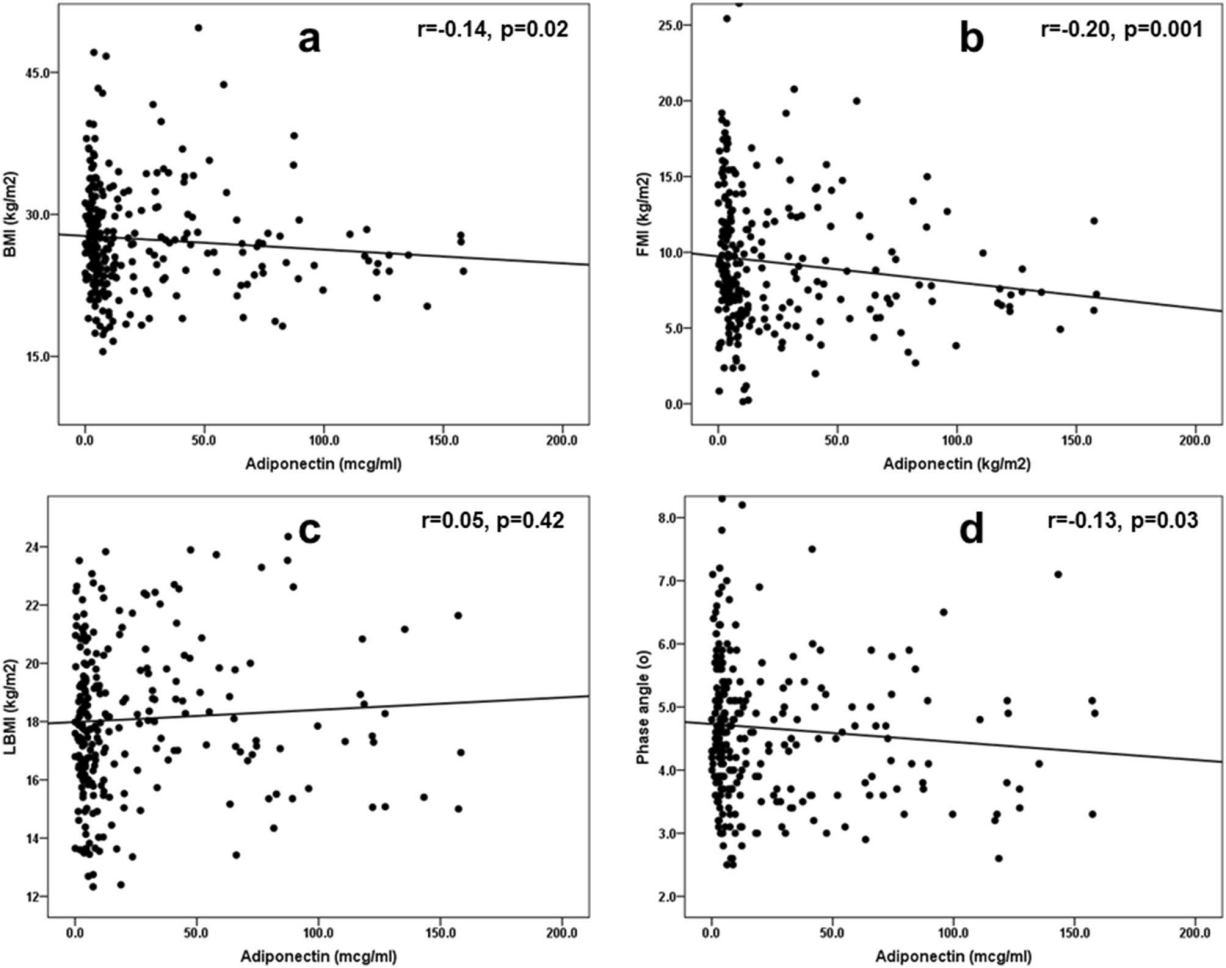
Spearman’s correlations between serum adiponectin (mcg/ml) and the body composition parameters [(**a**) BMI, (**b**) FMI, (**c**) LBMI, (**d**) phase angle] in the study population (n = 261). Abbreviations: BMI, body mass index; FMI, fat mass index; LBMI, lean body mass index.

**Figure 2:**
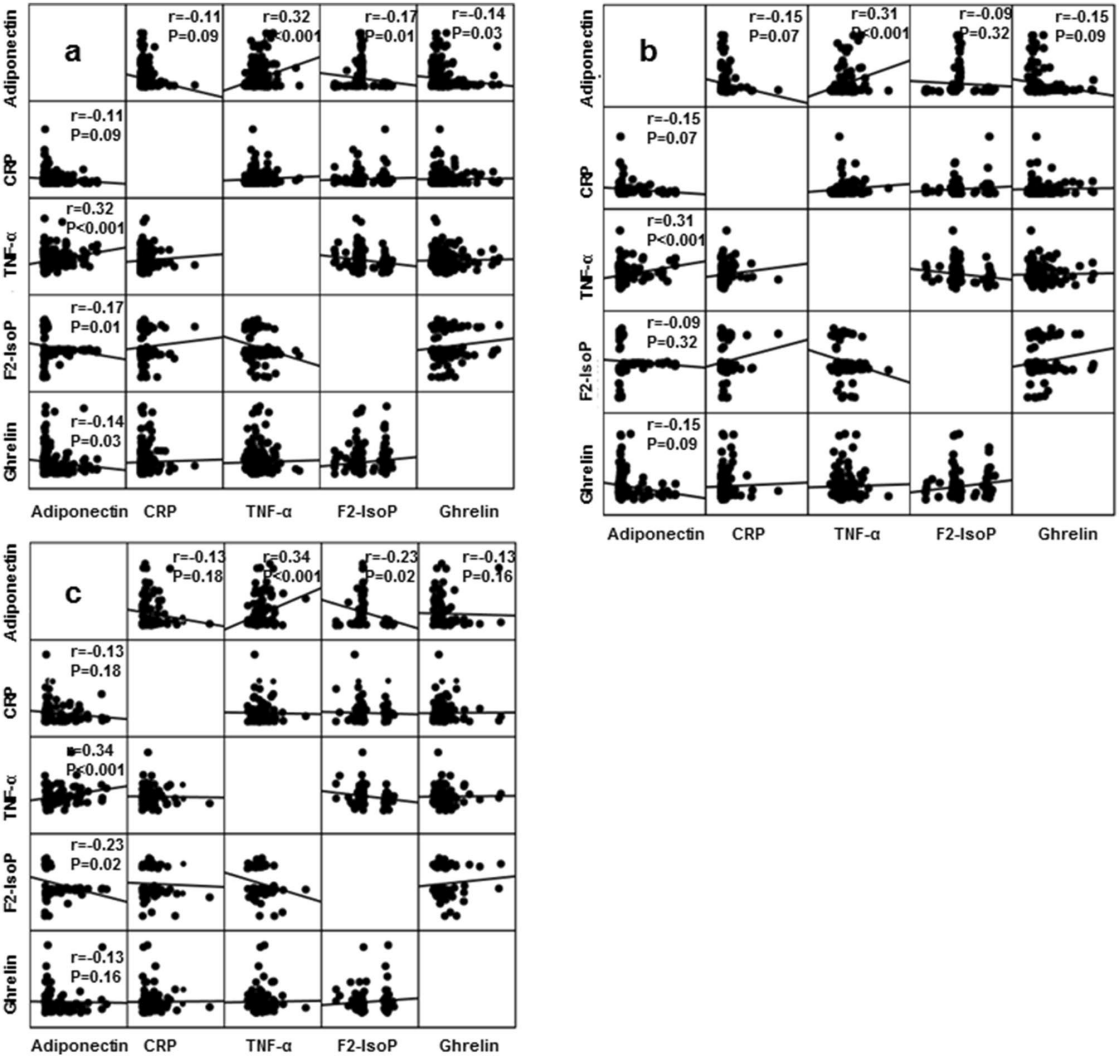
Correlation matrix for associations between serum adiponectin (mcg/ml) markers of inflammation, oxidative stress and appetite hormones in the whole population (n = 261) (**a**), a subgroup of patients with comorbidity index ≤ 4 (n = 142) (**b**), and a subgroup of patients with comorbidity index > 4 (n = 119) (**c**). Adjustments for age, sex, DM status, dialysis vintage, comorbidity index, residual renal function, smoking and Kt/V.

First, we studied the linear associations of adiponectin with body composition parameters and nutritional scores (malnutrition-inflammation score (MIS) and geriatric nutritional risk index (GNRI)) (Fig. 1). Adiponectin showed weak but statistically significant and negative correlations with BMI, FMI and phase angles (Figs. 1a,b,d, respectively) but not with lean body mass index (LBMI) (Fig. 1c). The weak and negative correlation of adiponectin with GNRI (r = − 0.14, *p* = 0.03) did not stand to further multivariable adjustments for age, gender, DM status, dialysis vintage, smoking, comorbidity index, residual renal function and Kt/V (data not shown). A correlation matrix (adjusted for the aforementioned demographic and clinical parameters) between adiponectin and inflammatory markers (CRP and TNF-α), oxidative stress (F2-IsoP) and appetite hormones (acyl-ghrelin), which expressed a linear relationship with adiponectin in Table 1, is shown in Fig. 2. Adiponectin was positively correlated to TNF-α and negatively correlated with CRP, F2-IsoP and acyl-ghrelin (Fig. 2a). When we examined the same associations separately in the groups of patients with high versus low comorbidity burden, a negative and significant correlation between adiponectin and F2-IsoP remained only in the group of patients with high comorbidities (Fig. 2c), whereas the association between adiponectin and TNF-α was not affected by this categorization (Figs. 2b,c). Because the significance of the correlations between adiponectin and CRP was borderline in our analyses, we decided to examine a nonlinear relationship between these variables using logistic regression analysis (Table 3). The inverse and significant correlation of adiponectin with CRP observed in the whole study population in multivariate models, presented the same direction of association and significance only in patients with a high comorbidity burden.

**Table 3:** Associations between serum adiponectin levels and inflammation defined as CRP ≥ 7.8 mg/L (above of the median of its distribution) in the whole study population and in subgroups of patients categorized by comorbidity index according to univariate and multivariable logistic regression analyses.

Variable	Univariate		Multivariable*	
	OR (95% conf. interval)	*p*	OR (95% conf. interval)	*p*
**All patients (n = 261)**				
CRP ≥ 7.8 mg/L				
Per 1 mcg/ml↑	0.99 (0.99–1.00)	0.05	0.99 (0.98–1.00)	0.03
≥ 8.28 mcg/ml	0.63 (0.39–1.04)	0.07	0.53 (0.31–0.90)	0.02
**Comorbidity index ≤ 4 (n = 142)**				
CRP ≥ 7.8 mg/L				
Per 1 mcg/ml↑	0.99 (0.99–1.01)	0.35	0.99 (0.98–1.00)	0.24
≥ 8.28 mcg/ml	0.70 (0.36–1.39)	0.31	0.61 (0.29–1.27)	0.19
**Comorbidity index > 4 (n = 119)**				
CRP ≥ 7.8 mg/L				
Per 1 mcg/ml↑	0.99 (0.98–1.00)	0.04	0.99 (0.07–1.00)	0.04
≥ 8.28 mcg/ml	0.48 (0.23–1.02)	0.06	0.44 (0.20–0.96)	0.04

CRP, C-reactive protein; OR, odds ratio; DM, diabetes mellitus.

*Adjusted for age, sex, DM status, dialysis vintage, residual renal function, comorbidity index, smoking and Kt/V.

**Figure 3:**
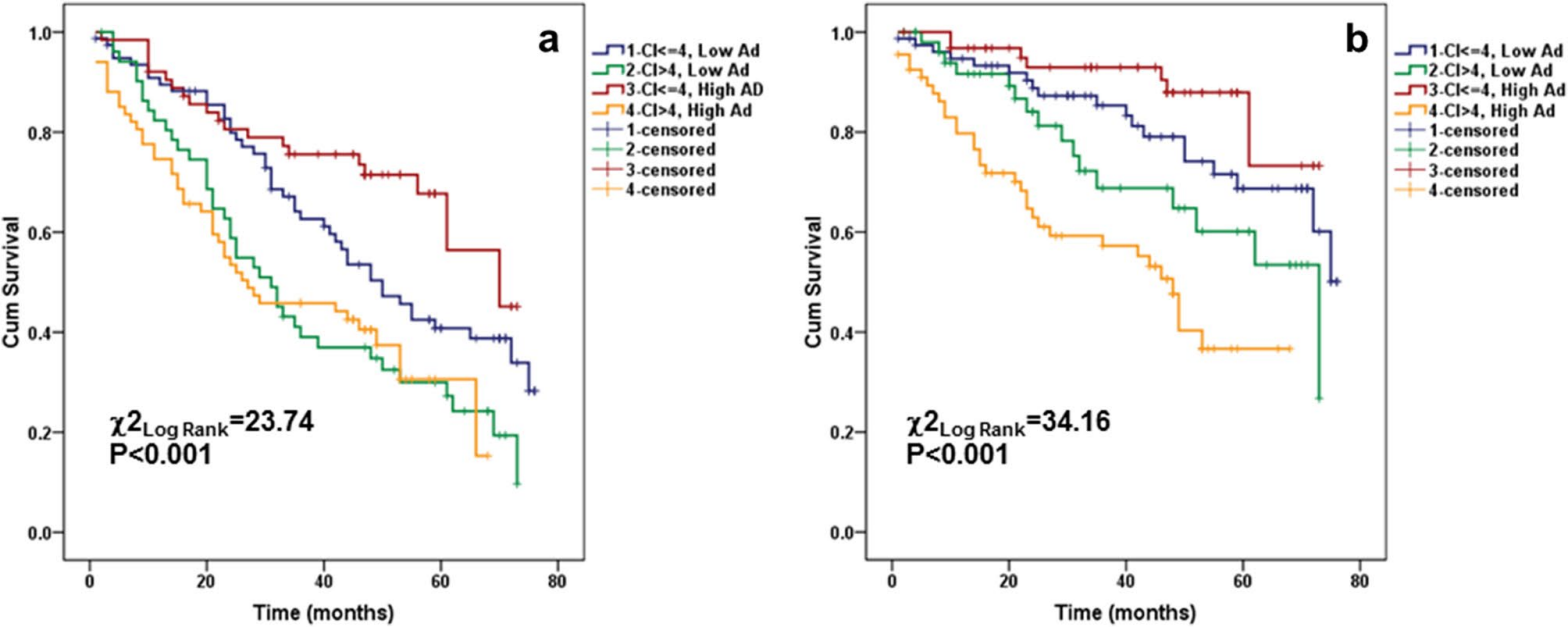
Kaplan–Meier survival curves of surviving patients comparing subgroups with the baseline serum comorbidity index and adiponectin categories (comorbidity index below and above median (4.0) and adiponectin below and above median (8.28 mcg/ml) values) cross-classified in 261 MHD patients followed for up to 6 years: (**a**) all-cause mortality, and (**b**) cardiovascular mortality.

Of the 64 patients in the low CI-high adiponectin group, 20 patients died (31.3%) compared to 43 out of 67 patients (64.2%) that died in the high CI-high adiponectin group during the follow-up period of the study. Moreover, 33 cardiovascular deaths (49.3%) occurred in the latter group versus seven cardiovascular deaths (11.0%) out of 64 patients that comprised the low CI-high adiponectin group. In survival analyses across the four CI-adiponectin categories, the group with low comorbidities and high adiponectin exhibited the best outcomes in all-cause mortality (log rank χ^2^ = 23.74, *p* < 0.001) (Fig. 3a). For cardiovascular mortality, the group with low comorbidities and high adiponectin exhibited the best outcomes as well, whereas in the high comorbidity group, conversely, high adiponectin expressed the association with the lowest survival rate (log rankχ^2^ = 34.16, *p* < 0.001) (Fig. 3b). Further data adjustment for age, gender, diabetes status, dialysis vintage, smoking, Kt/V and FMI did not substantially affect these results (Table 4). TNF-α together with F2-IsoP did not show a confounding effect on the associations of adiponectin with all-cause and cardiovascular mortality when added to multivariable models (Model 3, Table 3). But when TNF-α was replaced by CRP (Model 4, Table 3), the associations between adiponectin and mortality remained significant only in the low comorbidities with high adiponectin subgroup. The associations appeared to be identical to those shown in Model 4 when CRP was replaced by NLR or IL-6 (data not shown).

**Table 4:** Crude and adjusted all-cause and CVD-related mortality grouped according to CI and adiponectin levels^a^ in the study population (n = 261).

Variable	All-cause mortality		Cardiovascular mortality	
	HR (95% CI)	*p*	HR (95% CI)	*p*
**Low CI, low Ad (n = 78)**				
1.Crude	1.0	1.0		
2. Case-mix Adjusted	1.0	1.0		
3. 2 + TNF-α + F2-IsoP	1.0	1.0		
4. 2 + CRP + F2-IsoP	1.0 1.0			
**Low CI, high Ad (n = 64)**				
1.Crude	0.59 (0.35–1.00)	0.05	0.50 (0.21–1.19)	0.12
2. Case-mix Adjusted	0.45 (0.26–0.79)	0.005	0.36 (0.15–0.91)	0.03
3. 2 + TNF-α + F2-IsoP	0.44 (0.24–0.84)	0.01	0.32 (0.11–0.91)	0.03
4. 2 + CRP + F2-IsoP	0.45 (0.25–0.86)	0.02	0.33 (0.12–0.95)	0.04
**High CI, low Ad (n = 52)**				
1.Crude	1.71 (1.11–2.64)	0.02	1.67 (0.85–3.29)	0.14
2. Case-mix Adjusted	1.49 (0.92–2.43)	0.11	1.16 (0.55–2.41)	0.70
3. 2 + TNF-α + F2-IsoP	1.70 (1.02–2.84)	0.04	1.35 (0.62–2.94)	0.45
4. 2 + CRP + F2-IsoP	1.36 (0.65–2.85)	0.42	0.72 (0.25–2.06)	0.54
**High CI, high Ad (n = 67)**				
1.Crude	1.75 (1.14–2.69)	0.01	3.27 (1.81–5.90)	< 0.001
2. Case-mix Adjusted	1.52 (0.94–2.47)	0.09	2.43 (1.26–4.68)	0.008
3. 2 + TNF-α + F2-IsoP	1.88 (1.14–3.10)	0.01	2.73 (1.37–5.47)	0.004
4. 2 + CRP + F2-IsoP	1.43 (0.62–3.30)	0.40	1.33 (0.44–4.03)	0.62

Case-mix—adjusted for age, gender, diabetes status, dialysis vintage, residual renal function, smoking, Kt/V and FMI.

All variables included in the regression models are continuous except for categorical variables.

Abbreviations: CI, comorbidity index; HR, hazard ratio; CVD, cardiovascular disease; Ad, adiponectin; F2-IsoP, F2-isoprostanes.

^a^The group of patients who had low CI (defined as CI levels below median) and low adiponectin (defined as adiponectin levels below median) was used as a reference.

**Figure 4:**
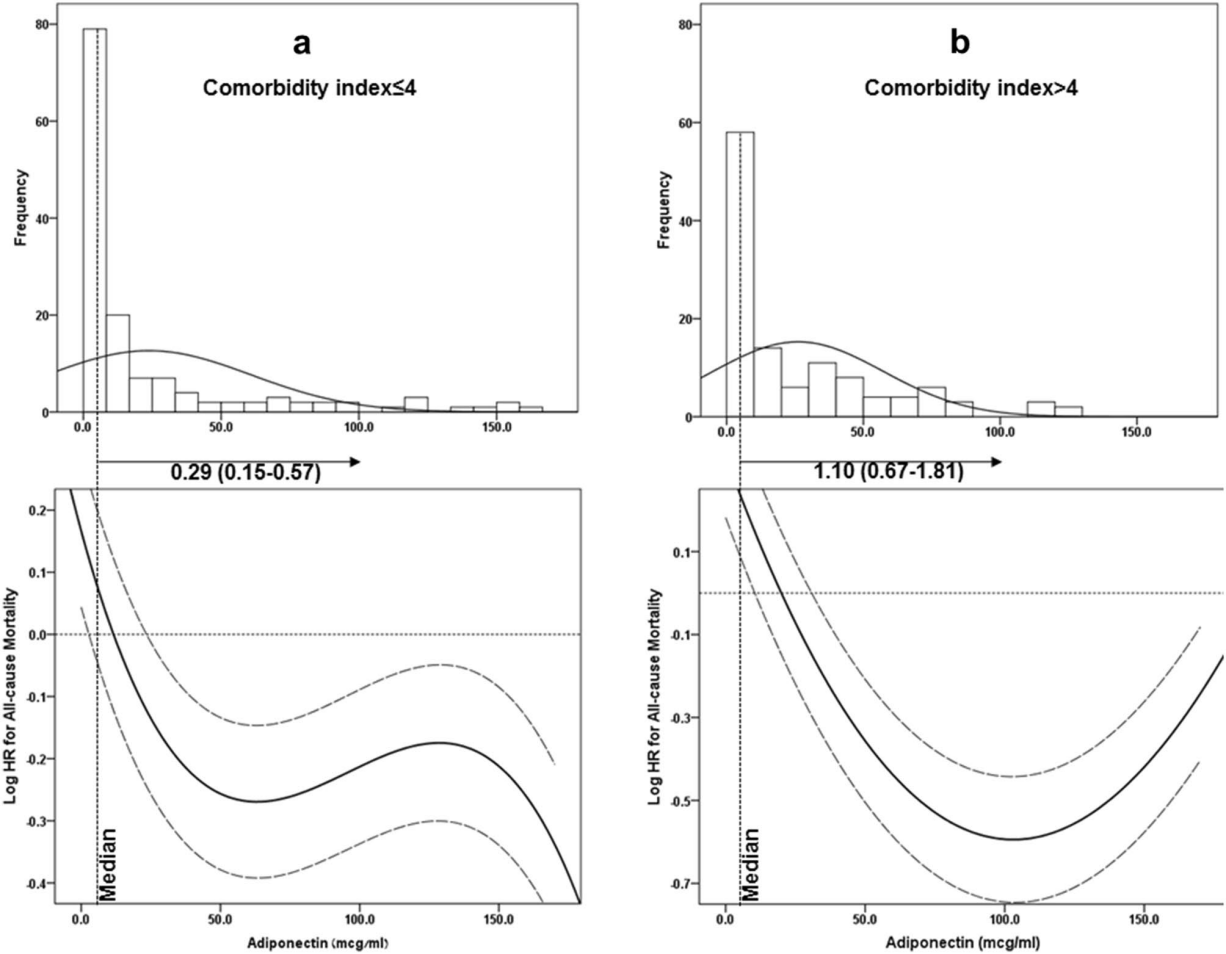
Histograms and multivariable-adjusted spline curves of adiponectin's association with all-cause mortality presented as log hazard ratios (solid lines) and 95% confidence interval (dashed lines) according to multivariable Cox regression models: (**a**), in MHD patients with CI ≤ 4; (**b**) in MHD patients with CI > 4. The models are plotted as restricted cubic splines with three internal knots. Multivariable models were adjusted for age, gender, DM status, co-morbidity index, smoking, residual renal function, dialysis vintage, Kt/V and FMI. Abbreviations: MHD, maintenance hemodialysis; CI, comorbidity index; FMI, fat mass index; DM, diabetes mellitus.

**Figure 5:**
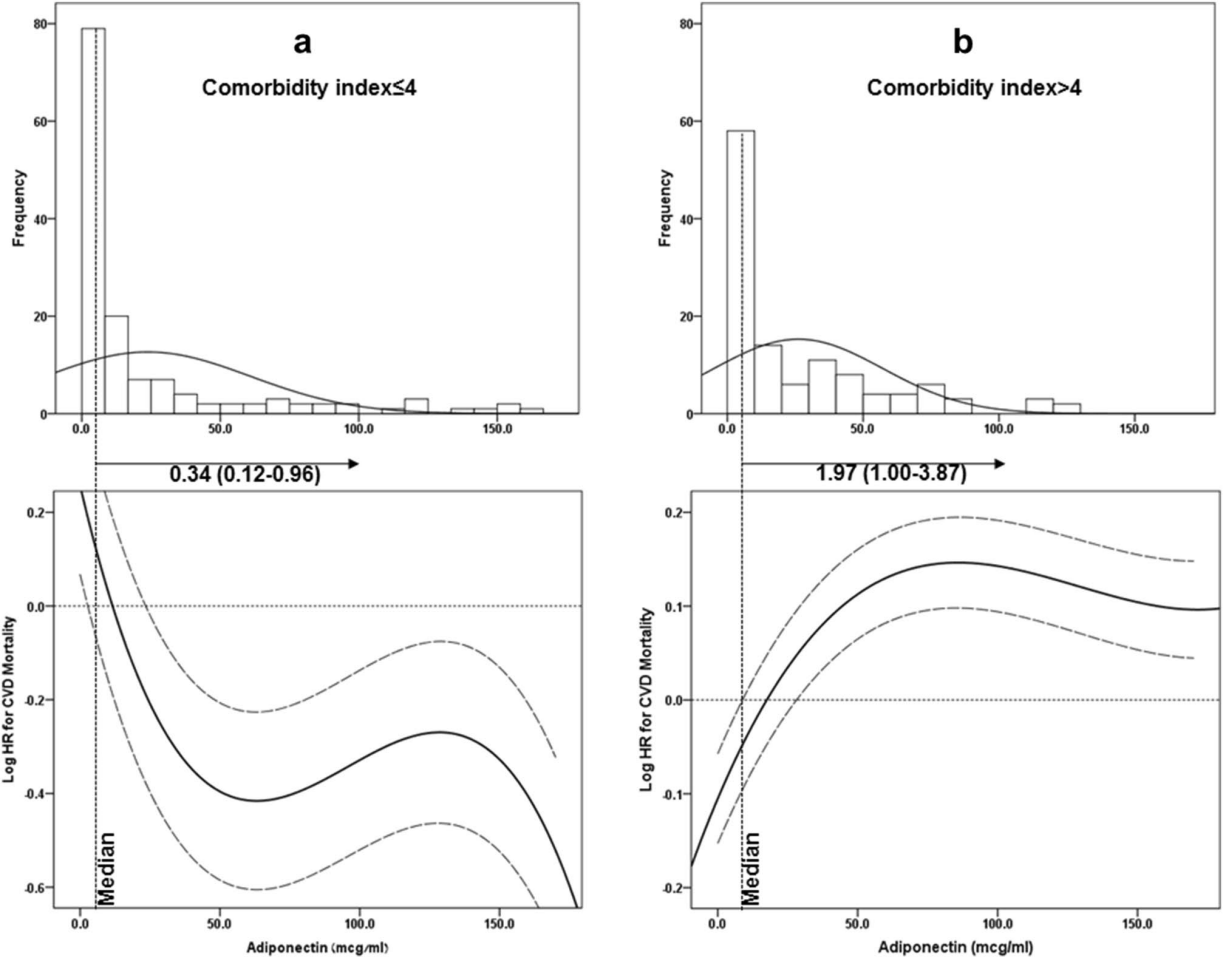
Histograms and multivariable-adjusted spline curves of adiponectin's association with cardiovascular mortality presented as log hazard ratios (solid lines) and 95% confidence interval (dashed lines) according to multivariable Cox regression models: (**a**), in MHD patients with CI ≤ 4; (**b**) in MHD patients with CI > 4. The models are plotted as restricted cubic splines with three internal knots. Multivariable models were adjusted for age, gender, DM status, co-morbidity index, smoking, residual renal function, dialysis vintage, Kt/V and FMI. Abbreviations: MHD, maintenance hemodialysis; CI, comorbidity index; FMI, fat mass index; DM, diabetes mellitus.

To visually demonstrate the directions of association between adiponectin levels and all-cause and cardiovascular mortality in our population with low versus high comorbidity, adiponectin was modeled by a restricted cubic spline with knots at the 5th, 45th, and 95th percentiles. When plotting adiponectin against the log hazard ratio for all-cause mortality and cardiovascular mortality, distinctive relationship patterns emerged for the participants with low versus high comorbidities. Specifically, in the MHD patient group with low comorbidities, increased adiponectin was associated with decreased all-cause mortality (Fig. 4a) and cardiovascular mortality (Fig. 5a). For the MHD patient group with high comorbidities, the relationship was U-shaped for all-cause mortality (Fig. 4b) and had a linear trend with the association of increased adiponectin with increased mortality for cardiovascular mortality (Fig. 5b).

## Discussion

In this study, we present a possible explanation for the paradoxical behavior of adiponectin as a marker of adverse clinical outcomes in MHD patients. Specifically, in MHD patients with few comorbidities, high levels of adiponectin are good prognostic markers, while in the very ill MHD population (i.e., MHD populations with a high comorbidity burden) high levels of adiponectin are associated with poor clinical outcomes. We found that adiponectin has an inverse relationship with inflammatory (CRP) and oxidative stress (F2-IsoP) markers only in MHD patients with severe comorbidities, when its positive association with TNF-α is independent of the comorbidity severity.

Overall, adiponectin’s behaviour was quite consistent with that reported in other MHD population studies in terms of its association with body composition, inflammatory indices and oxidative stress. First, our patients’ adiponectin levels were relatively high and comparable with those described in other MHD studies^8–10,16,17^. In our study, adiponectin showed a number of associations that have been reported previously in MHD patients, such as: inverse associations with BMI^17,21,24,25^, CRP^8,17,22^ and TNF-α^26^. The inverse association of adiponectin with oxidative stress markers (such as plasma malondialdehyde) has also been previously reported in the MHD population^16^. Therefore, our findings of the counter-associations between adiponectin and risk of mortality in our patients with mild comorbidities versus patients with severe comorbidities is of special interest. Unfortunately, none of the studies presenting the association between adiponectin and mortality in MHD patients reported any comorbidity score^8–10,17,21–23^. Instead, some of them stratified patients by reporting previous cardiovascular diseases^8,10,17,21–23^, DM rate^8–10,17,21,22^ and duration^17^. However, it is well known that because of the high prevalence of comorbidities in hemodialysis patients, simply accounting for a few broadly defined conditions may not be sufficient to control for case-mix differences between different MHD populations^27,28^. For this reason, it wasn't possible to examine differences between MHD populations in which a positive association of adiponectin with mortality was found compared to those in which the opposite association was described.

Large-scale studies in the general population could be used to compare the direction of adiponectin's association with mortality in patients with high comorbidities versus low comorbidities. In the largest population-based study of older adults (above 65 years of age) which examined the relationships of total and high-molecular-weight adiponectin with mortality among subgroups defined by baseline cardiovascular status, distinct associations of adiponectin with mortality were found^29^. The associations of adiponectin with all-cause and cardiovascular mortality shifted from a U-shaped relationship for participants free of prevalent clinical cardiovascular disease (including congestive heart failure) after extensively adjusting for potential confounders to a direct linear association for individuals with prevalent heart failure or atrial fibrillation. Further Kizer^30^ proposed the possible explanation for the interactions between adiponectin and comorbidities on mortality in elderly: low circulating adiponectin among younger adults, associated with obesity, low-grade inflammation and insulin resistance, predicts a higher risk of cardiovascular disease; by contrast, many comorbidities in elderly (including prevalent cardiovascular disease, heart failure and chronic kidney disease) may be expressed by elevated circulating adiponectin levels due to involuntary weight loss, increased production from non-adipose tissues or by direct stimulation by natriuretic peptides, or other mechanisms associated with high-grade inflammation. Such increased adiponectin levels are related to a higher risk of cardiovascular complications and mortality. In the 4,686 participants from the Lung Health Study cohort, serum adiponectin concentrations were inversely related to hospitalizations and mortality from coronary heart disease and cardiovascular disease, and were directly associated with increased risk of respiratory deaths^31^. Interestingly, in this study, smokers with mild to moderate airflow limitations were recruited while patients with almost all other comorbidities, including renal failure, were excluded. These studies only partially support our findings regarding the change in direction of the association between adiponectin levels and mortality risk in MHD patients depending on the severity of comorbidities. However, the question remains as to the mechanistic explanation for this behaviour of adiponectin.

High levels of adiponectin were associated with an increased risk of all-cause and CVD mortality in our severe comorbidities group. This can occur through one of the following two possible pathways: first, high levels of adiponectin in a uremic environment have detrimental effects that lead to higher mortality, or second, high adiponectin levels are a failed attempt of the body to overcome proinflammatory processes and/or oxidative stress in severe diseases including CVD, making adiponectin a marker of severe illness. The anorexigenic properties of adiponectin^32^ and its ability to accelerate energy expenditure^33^, the two actions related to the direct effect of adiponectin on the brain^32,33^, may associate high levels of adiponectin to low BMI and protein energy wasting. These two are well-known predictors of poor prognosis in MHD patients^34,35^. Although our study observed adiponectin's negative associations with ghrelin, a known orexogen^36^, as well as with BMI and with the nutritional status assessed by GNRI and phase angle, they did not explain the paradoxical association of adiponectin with mortality risk. Furthermore, significant associations with nutritional scores did not persist after multivariable adjustments. Additionally, neither modification effect of the comorbidities' severity on the relationship between adiponectin and ghrelin, nor a confounding effect of FMI on adiponectin's association to mortality risk were observed.

Adiponectin resistance is possibly due to an ineffective connection of adiponectin with downstream signal transductions, as has been previously described in experimental and clinical studies^37–40^. Indeed, simultaneous targeted disruption of both AdipoR1 and R2 in Lepr (-/-) mice abolishes adiponectin’s binding and actions, resulting in increased inflammation and oxidative stress, and leading to insulin resistance^37^. In a clinical setting, increased skeletal muscle adiponectin expression in congestive heart failure patients (fivefold higher than in healthy subjects) was characterized by downregulation of AdipoR1 that was most probably linked to deactivation of the peroxisome proliferator-activated receptor-alpha (PPAR-alpha)/AMP-activated protein kinase pathway^38^. Decreased expression levels of AdipoR1/R2 are also described in obesity^39^. Interestingly, adiponectin receptor expression was found to be upregulated by uremia in human tissues but accompanied by adiponectin resistance at the post-receptor level in ESKD patients^40^. Based on the above, we speculate that adiponectin levels rise as a defensive response to overcome proinflammatory and proatherogenic triggers, which translate into inverse associations of adiponectin with CRP and F2-IsoP in our severe comorbidity group. The less prominent proinflammatory influences in the low comorbidity group may explain the lack of association between adiponectin and markers of inflammation and oxidative stress.

One unique finding in our study was in the context of the inverse relationship between adiponectin and markers of oxidative stress. Although the negative correlation with plasma malondialdehyde levels has been previously described^16^, to the best of our knowledge, we are the first to report on the inverse association between adiponectin and F2-IsoP in MHD patients.

Another finding of our study is the positive association between adiponectin and TNF-α. This is somewhat surprising considering the anti-inflammatory properties of adiponectin. This association, however, has been described in MHD patients and possible explanations were recently discussed^26^. When TNF-α is associated with adiponectin (the study exposure) in our study similar to previous study^26^, the existing data linking elevated circulating TNF-α levels to all-cause and cardiovascular mortalities (the study outcome) in MHD patients is conflicting^19,41^. Although of borderline significance in association with adiponectin in our study, CRP (as well as IL-6 and NLR as inflammatory markers) compared to TNF- α is consistently related with all-cause and cardiovascular mortality in the literature^19,20,42,43^. The lack of relation to the outcome measure may explain why TNF- α as a covariate versus CRP (or IL-6 and NLR) did not express confounding effect in the association between adiponectin and mortality in our multivariate models.

Several limitations of the study should be taken into account. First, the study is observational in its nature, which does not allow for conclusions to be drawn about possible mechanisms. Second, the study was conducted at a single dialysis center and this prevents the generalizing of our findings to a wider MHD population. Third, the lack of markers of insulin resistance does not exclude the possibility of residual confounding on this basis. Finally, we did not measure high-molecular weight adiponectin, a fraction that has been reported to have stronger metabolic associations^1^. However, our research is strengthened by the rigorous, protocoled measurements of laboratory data, clinical measurements and comorbidities, prospective long-term follow up, the wide array of biochemical markers of nutrition, inflammation, oxidative stress, adipokines, and body composition parameters including anthropometrics and bioimpedance estimates.

In conclusion, we show that the changing direction of adiponectin’s prognostic associations to all-cause and cardiovascular mortality in MHD population depends on the severity of the underlying comorbidities. It is inverse in MHD patients with little comorbidity and direct in populations with many comorbidities. The adiponectin paradox is therefore a product of the study population selection, i.e., populations with a high level of comorbidities versus relatively healthy populations. The mechanism behind these relationships is possibly related to adiponectin’s uniqueness as an adipokine with a favourable cardiovascular profile that associates it with low all-cause and cardiovascular mortality in MHD patients with low baseline comorbidities. Conversely, in MHD patients with severe baseline comorbidities, adiponectin is disproportionally upregulated in an attempt to overcome the proatherogenic/inflammatory triggers and becomes a marker of a severe illness. The modification effect of the baseline comorbidities' severity on the association of adiponectin with CRP and F2-IsoP allow this assumption. Further epidemiologic and interventional studies are needed to better understand the adiponectin paradox on the mortality risk in the MHD population.

## Materials and methods

### Patients

This is a prospective observational study. The study was approved by our institution’s local ethics committee (Helsinki Committee, Shamir Medical Center). All research was performed in accordance with relevant guidelines/regulations and informed consent was obtained from all participants. The study included MHD patients on hemodialysis treatment for at least eight weeks, who were 18 years or older, and signed a local institutional review board approved consent form. Patients with an anticipated life expectancy less than six months (e.g., because of a metastatic malignancy) were excluded. In total, 261 patients undergoing MHD treatment at our outpatient HD clinic and at two satellite HD clinics (from the same region), were included in the study. The study population was described in more detail in a recent publication^44^. The patients were recruited from October 2010 through April 2012, and were followed until March 2017 or were censored (kidney transplantation or loss to follow-up).

The Kt/V values were calculated using the second generation Daugirdas's formula as recommended by DOQI guidelines (KDOQI guidelines (2000) for hemodialysis adequacy). At the start of the cohort, all study participants performed midweek interdialytic urine collection for measurement of urine output. Urine output was expressed as ml/24 h. Residual renal function (RRF) was defined as measured urine volume > 200 ml/day.

### Anthropometric measurements and handgrip strength

The following anthropometric variables were measured: body mass index (BMI), waist circumference (WC), triceps skinfold thickness (TSF), mid arm circumference (MAC), and calculated mid arm muscle circumference (MAMC). MAMC was estimated as follows:

$${\text{MAMC}}\,\left( {{\text{cm}}} \right)\, = \,{\text{mid}}\,{\text{arm}}\,{\text{circumference}}\,\left( {{\text{cm}}} \right)\, - \,0.{314} \times {\text{TSF}}\,\left( {{\text{mm}}} \right).$$

All measurements were made after dialysis when the patient was at dry weight (the right upper arm was used whenever possible, with exceptions made for patients in whom dialysis access placement, injury or stroke precluded measurement). The same trained dietitian performed all anthropometric measurements.

The patients performed handgrip strength (HGS) in both the dominant and nondominant arms using the Harpenden Handgrip Dynamometer (Yamar, Jackson, MI, USA). HGS was repeated three times and the highest value was noted.

### Nutritional scores

Overall nutritional assessment was performed using the malnutrition-inflammation score (MIS) and the geriatric nutritional risk index (GNRI).

Malnutrition-inflammation score (MIS) is described in detail in several previous studies^45,46^. It is a subjective global assessment (SGA) based method that consists of 10 components. The sum of all 10 components results in an overall score ranging from 0 (normal) to 30 (severely malnourished).

The GNRI index was calculated from the patient’s serum albumin and body weight by using the equation developed by Bouillanne et al.^47^:

Ideal weight in the present study was calculated from the Lorentz equations differently for men and women, as in the original GNRI equation^47^.

MIS and GNRI were previously shown to be a valid tool for longitudinal observations of MHD patient nutritional status^48^.

### Body composition analysis

Body composition was established by using body impedance analysis (B.I.A. Nutriguard-M, Data-Input, Frankfurt, Germany). We performed BIA within a half an hour post-dialysis according to the clinical application recommendations for analysis of bioelectrical impedance^49^. The BIA electrodes were placed on the non-access side of the patient and the patients were in a supine position for at least 5 min before the measurement. Resistance (R) and reactance (Xc), measured at 50 kHz, were used to calculate the phase angle by the following equation: phase angle (degrees) = arc-tangent (Xc/R) × (180/π). Recently, phase angle has been shown as a useful predictor for impaired muscle function, health-related quality of life, upcoming hospitalizations and mortality in MHD patients^50^.

The multifrequency technique (using three frequencies: 5, 50 and 100 kHz) were used to estimate the total body water (TBW), extracellular water (ECW), fat mass (FM) and lean body mass (LBM). These estimates were obtained using the NutriPlus software, version 5.1 (Data Input GmbH, Germany).

Fat mass and lean body mass were standardized by squared height (m^2^), and expressed in kg/m^2^ as fat mass index (FMI) and lean body mass index (LBMI), respectively.

### Comorbidity index and clinical outcomes

We calculated the comorbidity index, developed recently by Liu et al.^51^ and validated specifically for dialysis patient populations, as a measure of comorbid conditions. This index is defined by 11 comorbid conditions used by the United States Renal Data System (USRDS). The conditions are diabetes, ischemic heart disease, congestive heart failure, peripheral vascular disease, cerebrovascular disease (cerebrovascular accident/transient ischemic attack), dysrhythmia, other cardiac diseases (including pericarditis, endocarditis, myocarditis, other complications of heart disease, heart transplant, heart valve replacement, and cardiac devices), cancer, liver disease, gastro-intestinal bleeding, and lung disease (chronic obstructive pulmonary disease). Weights were assigned to each comorbid condition as follows: a weight of 1 was assigned to ischemic heart disease and diabetes; 3 to congestive heart failure; and 2 to each of the remaining comorbid conditions.

CVD was defined as myocardial infarction (MI) requiring coronary artery procedures such as angioplasty or surgery, cerebrovascular accident (CVA), or peripheral vascular disease (PVD) requiring angioplasty, bypass or amputation. Cardiovascular mortality was defined as death resulting from coronary heart disease, sudden death, stroke, or complicated peripheral vascular disease. Survival was determined from the day of examination.

### Laboratory evaluation

Predialysis blood samples and postdialysis serum urea nitrogen were obtained from non-fasting patients on a mid-week day. All biochemical analyses were measured by an automatic analyzer. Additionally, serum high sensitivity C-reactive protein (CRP) was measured by a turbidimetric immunoassay. Adiponectin, acyl-ghrelin, leptin, IL-6 and TNF-α levels were measured in plasma samples using commercially available enzyme-linked immunosorbent assay (ELISA) kits (R&D System, Minneapolis, MN, USA) according to the manufacturer’s protocol. F2-IsoP concentrations were measured by commercial ELISA kits (Cayman Chemical, 1180 E. Ellsworth Rd. Ann Arbor, MI. USA) in plasma samples collected in vacutainers containing EDTA.

### Statistical analysis

Data are expressed as means ± SDs for normally distributed data, medians and interquartile ranges (quartiles 1–3) for variables that did not follow a normal distribution, or frequencies for categorical variables.

To measure the differences between the variables in groups cross-classified by comorbidity index and adiponectin, a 2-factor MANOVA with Wilks-lambda was used. Since the dialysis vintage, comorbidity index, handgrip strength, TNF-α, CRP, IL-6, acyl-ghrelin, leptin and MIS levels were not normally distributed, these variables were log transformed (lg_10_) before they were inserted into this model.

Associations between two parameters were assessed using Pearson correlation coefficients or Spearman rank order correlation coefficients in cases of skewed distribution of data. Multivariate linear regression analyses were performed to obtain adjusted (partial) correlations. Additionally, univariate and multivariate logistic regressions were used to determine nonlinear associations between adiponectin and CRP in the study population.

Survival analyses were performed using the Kaplan–Meier survival curve and the Cox proportional hazard model. The univariate and multivariate Cox regression analyses are presented as (HR; Confidence Interval). All variables that have been hypothesized on theoretical grounds or that have been shown in previous studies to be confounders of the association between adiponectin and mortality were included as confounders in our multivariable models. Cox proportional hazard models with four incremental levels of covariate adjustment were constructed:

1. Unadjusted analyses (Model 1): No adjustment for covariates;
2. Case-mix–adjusted analyses (Model 2): Adjusted for age, gender, diabetes status, dialysis vintage, residual renal function, smoking, Kt/V and FMI.
3. Case-mix– and TNF-α and F2-IsoP adjusted analyses (Model 3); and
4. TNF- α replaced by CRP in Model 4.

Although Acyl-Ghrelin levels showed linear relationship with adiponectin and uric acid levels with comorbidity index according to Table 1, after adding them to model 3 (in Table 3), before including CRP in multivariate models, the significance and direction of the associations did not change. However, to avoid overadjustment bias, we decided not to include these models in Table 3.

The variation inflation factor (VIF) was measured for all multivariable models and no multicollinearity was detected (VIF resulted less than 1.5 for all models).

To further explore the association between adiponectin and survival, adiponectin was modeled by a restricted cubic spline with knots at the 5th, 45th, and 95th percentiles. This method allows to examine nonlinear associations as continuous mortality predictors as an alternative to inappropriate linearity assumptions^52^.

All statistical analyses were performed using SPSS software, version 18.0 (SPSS Inc, Chicago, IL).

## References

[1] Chandran M, Phillips SA, Ciaraldi T, Henry RR (2003). Adiponectin: More than just another fat cell hormone?. Diabetes Care.

[2] Gao H (2013). Evidence of a causal relationship between adiponectin levels and insulin sensitivity: A Mendelian randomization study. Diabetes.

[3] Ohashi K (2010). Adiponectin promotes macrophage polarization toward an anti-inflammatory phenotype. J. Biol. Chem..

[4] Okamoto Y (2002). Adiponectin reduces atherosclerosis in apolipoprotein E-deficient mice. Circulation.

[5] Szabó T (2014). Plasma adiponectin in heart failure with and without cachexia: Catabolic signal linking catabolism, symptomatic status, and prognosis. Nutr. Metab. Cardiovasc. Dis..

[6] Lindberg S (2015). interplay between adiponectin and pro-atrial natriuretic peptide and prognosis in patients with ST-segment elevation myocardial infarction. Am. J. Cardiol..

[7] Singer JR (2012). Adiponectin and all-cause mortality in elderly people with type 2 diabetes. Diabetes Care.

[8] Abdallah E, Waked E, Nabil M, El-Bendary O (2012). Adiponectin and cardiovascular outcomes among hemodialysis patients. Kidney Blood Press. Res..

[9] Rhee CM (2015). Association of adiponectin with body composition and mortality in hemodialysis patients. Am. J. Kidney Dis..

[10] Markaki A (2012). The role of serum magnesium and calcium on the association between adiponectin levels and all-cause mortality in end-stage renal disease patients. PLoS ONE.

[11] Wang Y (2006). Post-translational modifications of the four conserved lysine residues within the collagenous domain of adiponectin are required for the formation of its high molecular weight oligomeric complex. J. Biol. Chem..

[12] Bobbert T (2005). Changes of adiponectin oligomer composition by moderate weight reduction. Diabetes.

[13] Yamauchi T (2003). Cloning of adiponectin receptors that mediate antidiabetic metabolic effects. Nature.

[14] Markaki A, Psylinakis E, Spyridaki A (2016). Adiponectin and end-stage renal disease. Hormones (Athens).

[15] Elokely A, Shoukry A, Ghonemy TA, Atia M, Amr G (2012). Association of adiponectin with cardiovascular events in diabetic and non-diabetic hemodialysis patients. Saudi J. Kidney Dis. Transpl..

[16] Lim PS, Chen SL, Wu MY, Hu CY, Wu TK (2007). Association of plasma adiponectin levels with oxidative stress in hemodialysis patients. Blood Purif..

[17] Drechsler C, Krane V, Winkler K, Dekker FW, Wanner C (2009). Changes in adiponectin and the risk of sudden death, stroke, myocardial infarction, and mortality in hemodialysis patients. Kidney Int..

[18] Sasaki K (2021). Oxidative stress and inflammation as predictors of mortality and cardiovascular events in hemodialysis patients. The DREAM Cohort. J. Atheroscler. Thromb..

[19] Noori N (2011). Racial and ethnic differences in mortality of hemodialysis patients: role of dietary and nutritional status and inflammation. Am. J. Nephrol..

[20] Beberashvili I (2011). IL-6 levels, nutritional status, and mortality in prevalent hemodialysis patients. Clin. J. Am. Soc. Nephrol..

[21] Ohashi N (2008). Association of serum adiponectin levels with all-cause mortality in hemodialysis patients. Intern Med..

[22] Rao M, Li L, Tighiouart H, Jaber BL, Pereira BJ, Balakrishnan VS, HEMO Study Group (2008). Plasma adiponectin levels and clinical outcomes among haemodialysis patients. Nephrol. Dial. Transplant..

[23] Zhou Y, Zhang J, Zhang W, Ni Z (2016). Association of adiponectin with peripheral arterial disease and mortality in nondiabetic hemodialysis patients: Long-term follow-up data of 7 years. J. Res. Med. Sci..

[24] Tsigalou C (2013). Differential effect of baseline adiponectin on all-cause mortality in hemodialysis patients depending on initial body mass index. Long-term follow-up data of 4.5 years. J. Ren. Nutr..

[25] Delgado C (2017). Associations of body mass index and body fat with markers of inflammation and nutrition among patients receiving hemodialysis. Am. J. Kidney Dis..

[26] Ayerden Ebinç F (2009). The relationship between adiponectin levels and proinflammatory cytokines and left ventricular mass in dialysis patients. J. Nephrol..

[27] Miskulin D (2009). Key comorbid conditions that are predictive of survival among hemodialysis patients. Clin. J. Am. Soc. Nephrol..

[28] Goodkin DA (2003). Association of comorbid conditions and mortality in hemodialysis patients in Europe, Japan, and the United States: the Dialysis Outcomes and Practice Patterns Study (DOPPS). J. Am. Soc. Nephrol..

[29] Kizer JR (2012). Associations of total and high-molecular-weight adiponectin with all-cause and cardiovascular mortality in older persons: the Cardiovascular Health Study. Circulation.

[30] Kizer JR (2014). Adiponectin, cardiovascular disease, and mortality: parsing the dual prognostic implications of a complex adipokine. Metabolism.

[31] Yoon HI (2012). The complex relationship of serum adiponectin to COPD outcomes COPD and adiponectin. Chest.

[32] Coope A (2008). AdipoR1 mediates the anorexigenic and insulin/leptin-like actions of adiponectin in the hypothalamus. FEBS Lett..

[33] Qi Y (2004). Adiponectin acts in the brain to decrease body weight. Nat. Med..

[34] Ertilav M (2019). Impact of body mass index on short-term and long-term survival in prevalent hemodialysis patients. Hemodial. Int..

[35] Beberashvili I (2016). Geriatric nutritional risk index, muscle function, quality of life and clinical outcome in hemodialysis patients. Clin. Nutr..

[36] Pradhan G, Samson SL, Sun Y (2013). Ghrelin: much more than a hunger hormone. Curr. Opin. Clin. Nutr. Metab. Care.

[37] Yamauchi T (2007). Targeted disruption of AdipoR1 and AdipoR2 causes abrogation of adiponectin binding and metabolic actions. Nat. Med..

[38] Van Berendoncks AM (2010). Functional adiponectin resistance at the level of the skeletal muscle in mild to moderate chronic heart failure. Circ. Heart Fail..

[39] Kadowaki T, Yamauchi T (2005). Adiponectin and adiponectin receptors. Endocr. Rev..

[40] Martinez Cantarin MP, Keith SW, Waldman SA, Falkner B (2014). Adiponectin receptor and adiponectin signaling in human tissue among patients with end-stage renal disease. Nephrol. Dial. Transplant..

[41] Meuwese CL (2011). Trimestral variations of C-reactive protein, interleukin-6 and tumour necrosis factor-α are similarly associated with survival in haemodialysis patients. Nephrol. Dial. Transplant..

[42] Catabay C (2017). Lymphocyte cell ratios and mortality among incident hemodialysis patients. Am. J. Nephrol..

[43] Balboul Y (2020). Biological basis of lymphocyte ratios for survival prediction in hemodialysis patients: A longitudinal study. Int. Urol. Nephrol..

[44] Beberashvili I (2015). Serum uric acid as a clinically useful nutritional marker and predictor of outcome in maintenance hemodialysis patients. Nutrition.

[45] Kalantar-Zadeh K, Kopple JD, Humphreys MH, Block G (2004). Comparing outcome predictability of markers of malnutrition-inflammation complex syndrome in haemodialysis patients. Nephrol. Dial. Transplant..

[46] Rambod M (2009). Association of Malnutrition-Inflammation Score with quality of life and mortality in hemodialysis patients: A 5-year prospective cohort study. Am. J. Kidney Dis..

[47] Bouillanne O (2005). Geriatric Nutritional Risk Index: A new index for evaluating at-risk elderly medical patients. Am. J. Clin. Nutr..

[48] Beberashvili I (2013). Comparison analysis of nutritional scores for serial monitoring of nutritional status in hemodialysis patients. Clin. J. Am. Soc. Nephrol..

[49] Kyle UG (2004). ESPEN: Bioelectrical impedance analysis—Part II: Utilization in clinical practice. Clin. Nutr..

[50] Beberashvili I (2014). Bioimpedance phase angle predicts muscle function, quality of life and clinical outcome in maintenance hemodialysis patients. Eur. J. Clin. Nutr..

[51] Liu J, Huang Z, Gilbertson DT, Foley RN, Collins AJ (2010). An improved comorbidity index for outcome analyses among dialysis patients. Kidney Int..

[52] Howe CJ (2011). Splines for trend analysis and continuous confounder control. Epidemiology.

